# The complete chloroplast genome sequence of the threatened *Cypripedium calceolus* (Orchidaceae)

**DOI:** 10.1080/23802359.2019.1693933

**Published:** 2019-11-22

**Authors:** Li-Jie Zhang, Rui Ding, Wei-Wei Meng, Hua-Lei Hu, Xu-Hui Chen, Hai-Hong Wu

**Affiliations:** a College of Forestry, Shenyang Agricultural University, Shenyang, China;; b College of Land and Environment, Shenyang Agricultural University, Shenyang, China;; c College of Bioscience and Biotechnology, Shenyang Agricultural University, Shenyang, China;; d Institute of Floriculture, Liaoning Academy of Agricultural Sciences, Shenyang, China

**Keywords:** Chloroplast genome, *Cypripedium calceolus*, rare orchid, conservation

## Abstract

The complete chloroplast genome of *Cypripedium calceolus*, a rare species in the family Orchidaceae was reported in this study. The genome size is 175,122 bp in length, and contains four sub-regions: 97,486 bp of large single copy (LSC) and 22,260 bp of small copy (SSC) regions, separated by 27,688 bp of inverted repeat (IR) regions. A total of 133 genes were annotated, including 87 protein-coding genes, 38 tRNA genes and 8 rRNA genes. The GC content of this cp genome is 34.36%. Phylogenetic analysis revealed a close relationship between *C. calceolus* with *C. japonicum* and *C. formosanum*. This is the first complete cp genome for *C. calceolus* that would be useful for conservation and phylogenetic studies of this species.

*Cypripedium* (Cypripedioideae, Orchidaceae) is a remarkable genus with highly ornamental value because of their showy flowers. *Cypripedium calceolus* is a terrestrial orchid in this genus and naturally distributed in northeast China, Japan, North Korea and Europe (Lang et al. 1999). However, due to habitat destruction and ruthless collection for horticultural purposes (Minasiewicz et al. 2018; Fay 2018), the range of *C. calceolus* is declining rapidly in recent years. As a result, *C. calecolus* is now regarded as a least concern species according to the IUCN (Rankou and Bilz 2014), and is listed among the level-I state protected wild plants by the Chinese Central Government. In this study, we assembled and characterized the complete chloroplast (cp) genome of *C. calecolus* for the first time which will contribute to development of conservation strategy for this rare orchid species. The annotated cp genome sequence was submitted to GenBank under the accession number of MN602053.

The plant material of *C. calecolus* was collected from Longgang mountain (N41°58′09″, E125°08′56″), Liaoning, northeast China. The specimen was stored with the archival number of ORCHID_CYP_CAL_01 at College of Forestry of Shenyang Agricultural University. Total genomic DNA was extracted and sequenced on the Illumina Miseq sequencing platform by Shanghai Personal Biotechnology Co. Ltd, China. A reference-guided assembly was used to reconstruct the cp genome, with *C. japonicum* (KJ625630) as the reference. The complete genome was manually annotated by comparing with the cp genomes of *Cypripedium* species (Kim et al. 2014; Luo et al. 2014; Lin et al. 2015).

The cp genome of C. *calecolus* is 175,122 bp in length and has a typical circular structure including a large single-copy (LSC) region of 97,486 bp and a small single-copy (SSC) region of 22,260 bp, which is separated by a pair of inverted repeat (IR) regions of 27,688 bp. The overall GC content is 34.36% and in the LSC, SSC, and IR regions are 31.57%, 26.13%, and 42.58%, respectively. A total of 133 genes were successfully annotated, including 87 protein-coding genes, 38 tRNA genes and 8 rRNA genes. Seven protein-coding, eight tRNA, and all four rRNA genes were duplicated in IR regions. In total, 22 intron-containing genes were annotated, with 4 genes having two introns and the rests having one intron.

To study the phylogenetic position of C. *calecolus* within the Orchidaceae family, the published cp genomes of 46 species from this family were selected for analysis. The complete genomes were downloaded from the NCBI GenBank database and were aligned using HomBlocks (Bi et al. 2018) with the Gblocks method (Talavera and Castresana 2007). Phylogenetic analysis was performed by maximum-likelihood analysis with MEGA 7.0 (Kumar et al. 2016). The topology of the phylogenetic tree corroborates the current taxonomy of the family Orchidaceae at the subfamily level (Cameron et al. 1999), and C. *calecolus* is closely related to the congeneric *C. japonicum* and *C. formosanum*. The complete cp genome information provided data useful for conservation works on *C. calecolus* as well as for phylogenetic studies within Orchidaceae.

## Figures and Tables

**Figure 1.**
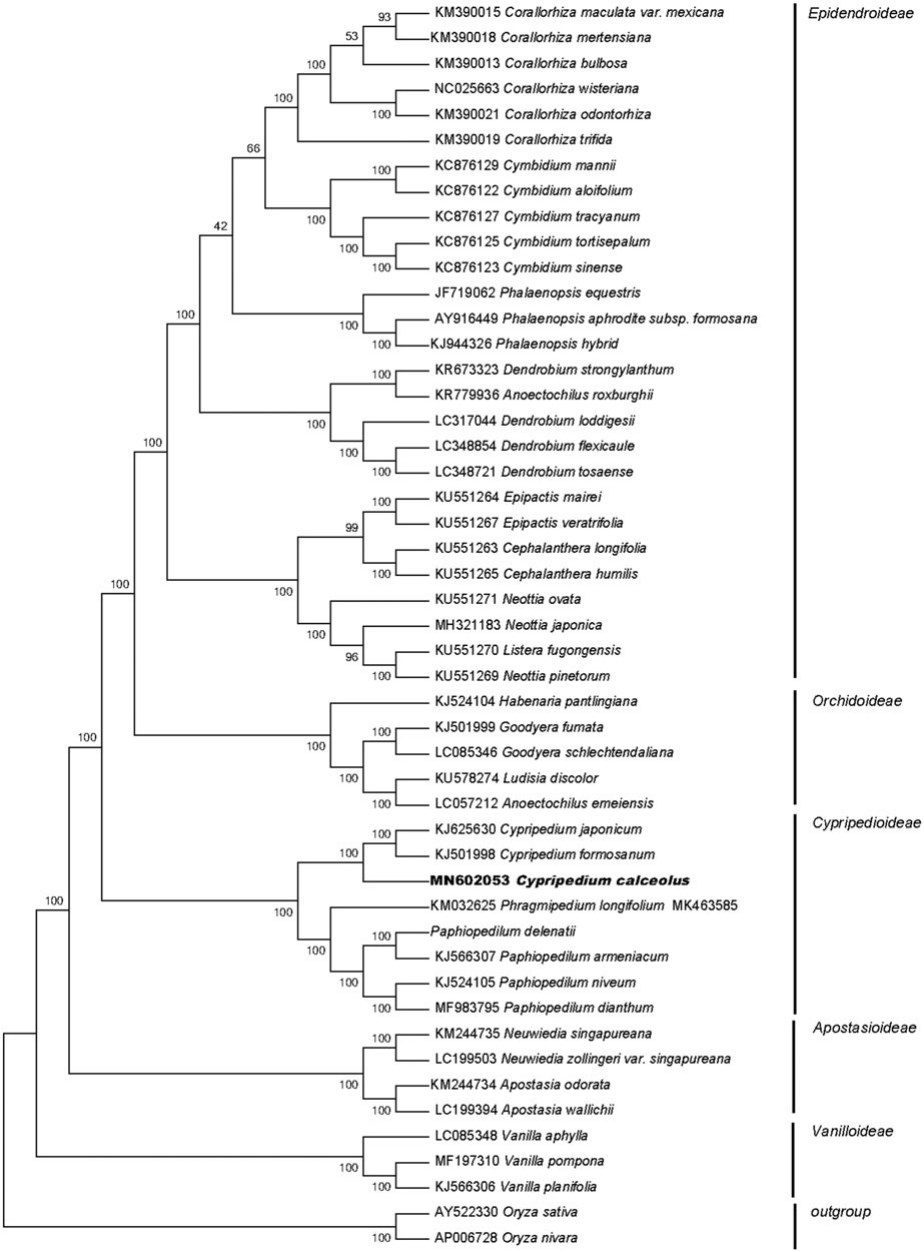
Phylogenetic raltionship of 47 orchids species built by maximum-likelihood method based on complete chloroplast genomes, with Oryza genus As the outgroup. The branch support was determined by computing 1000 non-parametric bootstrap replicates.
